# WHO/ISUP classification, grading and pathological staging of renal cell carcinoma: standards and controversies

**DOI:** 10.1007/s00345-018-2447-8

**Published:** 2018-08-19

**Authors:** Anne Y. Warren, David Harrison

**Affiliations:** 1 Department of Histopathology, Cambridge University Hospitals NHS Foundation Trust, Cambridge, CB2 0QQ UK; 2 School of Medicine, University of St Andrews, St Andrews, KY16 9TF UK

**Keywords:** Renal cell carcinoma, Pathology, Grading, Staging, Review

## Abstract

**Purpose:**

Pathological parameters assessed on biopsies and resection specimens have a pivotal role in the diagnosis, prognosis and management of patients with renal cell carcinoma (RCC).

**Methods:**

A non-systematic literature search was performed, updated to January 2018, to identify key standards and controversies in the pathological classification, grading and staging of RCC.

**Results:**

Although most RCCs exhibit characteristic morphology that enables easy categorisation, RCCs show considerable morphological heterogeneity and it is not uncommon for there to be difficulty in assigning a tumour type, especially with rarer tumour subtypes. The differentiation between benign and malignant oncocytic tumours remains a particular challenge. The development of additional immunohistochemical and molecular tests is needed to facilitate tumour typing, because of the prognostic and therapeutic implications, and to enable more reliable identification of poorly differentiated metastatic tumours as being of renal origin. Any new tests need to be applicable to small biopsy samples, to overcome the heterogeneity of renal tumours. There is also a need to facilitate identification of tumour types that have genetic implications, to allow referral and management at specialist centres. Digital pathology has a potential role in such referral practice.

**Conclusion:**

Much has been done to standardise pathological assessment of renal cell carcinomas in recent years, but there still remain areas of difficulty in classification and grading of these heterogeneous tumours.

## Introduction

Pathological parameters assessed on biopsies and resection specimens have a pivotal role in the diagnosis, prognosis and management of patients with renal cell carcinoma (RCC). Standardisation of specimen handling and pathological assessment is, therefore, critical to ensuring that the information contained within histopathology reports is accurate and consistent, for both diagnostic and research purposes. Although various local and national guidelines have been utilised in clinical practice for many years, several recent initiatives have done much to improve consistency at an international level, enabling pathologists to provide the high-quality information required. In 2016, the WHO published a fourth edition of its classification of urological tumours, which is the current internationally recommended system for typing of renal tumours [1]. The updated version of this ‘Blue book’ also provides epidemiological, clinical and pathological information on the wide range of renal tumours that may be encountered in clinical practice.

## Methods

A non-systematic literature search was conducted using Medline. The reference lists of selected manuscripts were checked manually for eligible articles. The most contemporary guidelines and relevant articles are included, updated to January 2018.

## Results

### Best practice guidelines

In 2012, the European Network of Uropathology (ENUP) published a survey of the practice of handling nephrectomy specimens by pathologists from 15 European countries [2]. This was followed in the same year by an International Society of Urological Pathology (ISUP) consensus conference on renal tumour pathology in Vancouver. Members of the Society were asked to complete a pre-meeting questionnaire on all aspects of their practice of specimen handling and pathological reporting of renal tumours. The results were presented at the conference by a panel of experts and were discussed and voted upon to determine what should be considered best practice. Following this conference, a series of ISUP publications has provided valuable guidance on specimen handling, tumour typing, grading and assessment of prognostic factors [3–8]. Subsequently, ISUP guidance for pathologists on best practice for use of routine immunohistochemistry in the assessment of renal cell tumours has also been published [9, 10].

In addition, the International Committee for Cancer Reporting (ICCR) has devised a series of international datasets for pathologists reporting tumour pathology, with the renal tumour datasets published in 2017 [11, 12]. In the UK, the third edition of the Royal College of Pathologists dataset for renal cancer reporting was published in 2017 [13]. Similar protocols are readily available from, for example, the College of American Pathologists (www.cap.org) and the Royal College of Pathologists of Australasia (www.rcpa.edu.au). These datasets contain guidance on which evidence-based ‘core’ pathological data items should always be included in histopathology reports, to provide the essential information required for patient management. Other items are also recommended for reporting, but are considered ‘non-core’ optional data at present due to insufficient evidence of their prognostic significance.

### Pathological parameters and stage

The main core items included in renal tumour pathology reports, currently deemed essential for patient management, are discussed below.

#### Tumour type

The 2016 WHO classification of renal tumours is based on a combination of morphological, molecular and genetic features [1]. RCCs represent the most common renal tumour in adults and are divided into a number of different histological types. The most common is the clear cell type (70–90%), followed by papillary (10–15%) and chromophobe RCCs (3–5%). Many studies, including large multicentre studies, have shown that tumour type has prognostic significance [6, 14–20]. Tumour type also has utility in selection of patients for adjuvant therapy, further underlining the importance of assigning tumours to the correct category on pathological assessment [21]. The major tumour types are discussed below, with their key pathological features, as well as rarer tumours that have differing clinical significance and may cause diagnostic difficulty.

*Clear cell RCC* has a worse prognosis than papillary or chromophobe RCCs, when matched for stage, and is more likely to present at an advanced stage or with existing metastases [20, 22–24]. In 90% of cases, these tumours exhibit alterations in the von Hippel–Lindau tumour suppressor (VHL) gene on chromosome 3 [25]. Most tumours are sporadic, but multiple bilateral tumours are seen in von Hippel–Lindau syndrome, a rare autosomal dominant condition also associated with a variety of other tumours that include haemangioblastomas of the retina and central nervous system [26]. Multifocal sporadic tumours are rare and a recent study has shown that apparent multifocality may be due to retrograde venous invasion from a single tumour [27]. Grossly, clear cell RCCs characteristically contain solid yellow areas with variable amounts of cystic change, haemorrhage and necrosis. Although on microscopy they are classically composed of clear cells set within a fine intricate vascular network, they may consist entirely of cells with eosinophilic granular cytoplasm, particularly if high grade. On immunohistochemistry they characteristically co-express pan-cytokeratin and vimentin and are carbonic anhydrase IX (CA-IX) positive, but are usually Cytokeratin 7 (CK7) negative. The multilocular cystic renal neoplasm of low malignant potential was formerly included in the clear cell carcinoma category, but is now known to have indolent behaviour and an excellent prognosis, regardless of size, with no reported metastases [6, 28]. It is rare (< 1% of renal tumours), but is readily diagnosed if morphological criteria are strictly adhered to [1]. These cystic tumours characteristically have thin fibrous septae containing low-grade clear cells, but no solid expansile clear cell nodules that are seen in clear cell RCC. They have been shown to have chromosome 3p deletions and VHL gene mutations similar to clear cell RCCs [1]. Correct pathological diagnosis of these tumours is important, as they may be managed conservatively.

*Papillary RCCs* are grossly solid, with or without cystic change or encapsulation, and are often grey or brown in colour with a soft friable cut surface showing frequent necrosis and haemorrhage. On microscopy, according to the current WHO classification, they are divided into type 1 or type 2 tumours, determined primarily by their differing cytological features, and mixed patterns occur [1, 6, 29]. An oncocytic variant (composed of cells with abundant eosinophilic/pink cytoplasm) has also been described morphologically, but is included under the general category of papillary RCC in this classification system [1, 18].

Type 1 papillary RCCs usually consist of papillary structures lined by cuboidal cells with low-grade nuclei. Collections of foamy macrophages are often present within the papillary fibrovascular cores and calcifications (psammoma bodies) and intracellular haemosiderin are common. They may also show solid growth, with very compact papillary structures. The tumours have a typical profile on immunohistochemistry, including strong CK7 and alpha-methylacyl-CoA racemase (AMACR) expression and at most focal CA-IX expression. At the molecular level, type 1 tumours typically show gains in chromosomes 7 and 17, and Y chromosome loss. Most tumours are sporadic, but there are familial cases in the autosomal dominant hereditary papillary RCC syndrome, where germline MET proto-oncogene mutations on chromosome 7 result in multiple bilateral tumours [30]. Extrarenal manifestations are not a feature of this familial syndrome [30]. Type 1 tumours generally present with a lower grade and stage at diagnosis and have a better outcome than type 2 tumours [31]. Tumours with the histological appearance of a non-encapsulated type 1 tumour and up to 15 mm in size are classified as papillary adenomas, rather than carcinomas, because they show benign clinical behaviour [1].

Morphologically, type 2 tumours have cells with more abundant eosinophilic cytoplasm that show nuclear pseudostratification and higher grade nuclei. They also show more variable protein expression on immunohistochemistry than type 1 tumours, often including loss of CK7. At the molecular level, these tumours are associated with NRF2–ARE pathway activation and can be divided into several distinct molecular subtypes that are associated with differing patient survival. Papillary RCCs are now known to represent a much more heterogeneous group of tumours than implied by the 2016 WHO classification [32, 33]. A recent study by Saleeb et al. suggests that using a combination of morphological features, immunoprofiles and molecular analysis, papillary RCCs can be divided into four subtypes and that this type of grouping would be a better means of guiding patient management [34].

Papillary RCC is more often multifocal and bilateral than the other common tumour types, as seen in approximately 10% of cases [6]. Papillary RCCs are also more frequent in acquired cystic kidney disease.

A less common tumour that may show overlapping morphology with type 1 papillary RCCs, particularly in limited biopsy samples, is the mucinous tubular and spindle cell carcinoma (MTSCC) [1]. This is classically composed of elongated tubules and spindle cells, both cytologically low grade, and abundant intercellular mucin. It has a similar immunohistochemical profile to papillary RCCs, adding to the difficulty in distinguishing these tumours in some cases. Ren et al. have, however, demonstrated that MTSCCs show multiple chromosome losses and lack trisomy 7 and 17, enabling separation on molecular studies [35]. These tumours are more common in females and generally exhibit indolent behaviour, but distant metastases have been reported [1, 18].

*Chromophobe RCC* is usually sporadic and generally has a good prognosis. Most of these tumours are confined to the kidney at diagnosis, though they may be large at the time of presentation [36]. They are characteristically tan in colour, similar to the benign renal oncocytoma that is the main differential diagnosis. On microscopy, chromophobe RCCs characteristically consist of large cells with prominent cell membranes, pale cytoplasm and crinkled ‘raisinoid’ nuclei with perinuclear halos. An eosinophilic variant also occurs, where the cells have an oncocytic cytoplasmic appearance and the nuclear features described are often less apparent. Chromophobe RCCs are characterised by multiple chromosome copy number alterations [37]. Hybrid oncocytic-chromophobe tumours, with a mixed morphology, may occur sporadically or in the Birt–Hogg–Dubé syndrome. The latter shows autosomal dominant inheritance and is associated with FLCN gene mutations on chromosome 17 [6, 18, 26]. There are associated cutaneous lesions, pulmonary cysts and spontaneous pneumothoraces [30]. The hybrid renal tumours are usually small and show indolent behaviour and are amenable to conservative management [38]. A variety of other renal tumour types have also been reported in this syndrome [30].

*Collecting duct carcinoma* is a rare (1–2%) and highly aggressive type of RCC arising in the renal medulla. It may be difficult to distinguish histologically from urothelial carcinoma of the renal pelvicalyceal system, due to similar infiltrative high-grade variable morphology and their overlapping immunohistochemistry profiles. Distinction from metastatic tumours may also be problematic, particularly adenocarcinomas, and diagnosis is by exclusion of other entities. Metastatic disease is common at the time of diagnosis and the majority of patients do not survive 2 years from diagnosis [1, 18]. Renal medullary carcinoma has similar morphology and occurs in association with sickle cell trait or disease. This is rare, aggressive and occurs more often in younger adults. In contrast to collecting duct carcinomas, these tumours may express OCT3/4 on immunohistochemistry and show loss of expression of SMARCB1 (INI1) [1, 39].

*MiT family translocation RCCs* are rare and should be considered particularly in children and young adults presenting with RCC, although they also occur in the adult population [40]. They result from gene fusions involving the MiT transcription factor genes TFE3 and TFEB, with differing fusion partners. The best morphologically described tumours of the group are those associated with Xp11 and *t*(6;11) translocations. The former may be recognised by their distinctive clear cell morphology with voluminous cells, and a papillary architecture, sometimes with frequent calcifications (psammoma bodies). The less common *t*(6;11) translocation tumours have a characteristic biphasic pattern with distinct groups of large and small epithelioid cells. The MiT family translocation RCCs commonly show weak expression of epithelial markers on immunohistochemistry and some express melanoma markers and cathepsin-K. Diagnosis requires fluorescence in situ hybridisation (FISH) to confirm the presence of the translocation, as use of immunohistochemistry has proven technically challenging. They may exhibit aggressive clinical behaviour, particularly in adults or those with the Xp11 translocation, and tend to develop early nodal metastases [1, 18, 36, 41]. They may be erroneously classified pathologically as clear cell RCCs, particularly when occurring in the adult population. This is of clinical significance, as they may not respond to the vascular endothelial growth factor (VEGF)-targeted treatments.

*Newly recognised types* A number of provisional tumour types recognised at the 2012 ISUP Vancouver consensus meeting have been included as separate entities in the 2016 WHO classification, as their morphology, immunoprofile and molecular characteristics are now better understood [1, 6]. These include the clear cell papillary RCC that shows indolent behaviour, with no reported local recurrences or metastases, but would have been previously diagnosed as a clear cell RCC [42]. These are usually small tumours and are often cystic with compact tubulo-papillary solid areas. A distinctive morphological feature is the linear orientation of tumour cell nuclei away from the basement membranes, although a similar appearance may be seen focally in clear cell RCCs. On immunohistochemistry they are strongly CK7 positive, unlike most clear cell RCCs, high molecular weight cytokeratin positive, AMACR negative and exhibit a distinct ‘cup-shaped’ staining pattern with CA-IX. Molecular studies show them to be distinct from clear cell and papillary RCCs [1]. They occur sporadically or in association with acquired cystic renal disease and are now recognised as being the fourth most common type of renal cell carcinoma [42]. Another tumour occurring more frequently in the clinical setting of acquired cystic kidney disease is the acquired cystic kidney disease-associated RCC. These tumours usually show indolent behaviour unless exhibiting high-grade features. They often appear to arise within a cyst and are commonly multifocal and bilateral. Characteristic morphological features are a ‘sieve-like’ architecture and the presence of abundant oxalate crystals [1]. They are typically AMACR positive, but CK7 and CA-IX negative. They frequently occur with other renal tumour types [43].

*Tubulocystic RCC* is another rare, usually indolent, good prognosis tumour type more frequently seen in men. It was thought to be related to papillary RCCs, but is now accepted as a separate entity [1, 44]. It has a characteristic ‘bubble wrap’ appearance grossly, due to the presence of fibrotic stroma separating cystic spaces. Small tubules are present within the stroma microscopically and are lined by cells with eosinophilic cytoplasm and round nuclei with nucleoli of variable prominence. The tumour cells may also have a ‘hobnail’ appearance [1, 18]. These tumours express AMACR and CK7 on immunohistochemistry. On molecular analysis, there have been conflicting reports, but in recent series of RCCs with a pure tubulocystic morphology, trisomy of chromosomes 7 and 17 observed in papillary RCCs was not present and these tumours have also been shown to have a molecular signature distinct from the more common RCC tumour types [45–47]. Other tumours may show areas with a tubulocystic pattern, including papillary RCCs, hereditary leiomyomatosis renal cell carcinoma-associated RCC, the MiT family translocation RCCs, collecting duct carcinoma and unclassified RCCs, causing diagnostic difficulty, but such tumours with mixed morphology are not included in this tumour category.

*Hereditary tumours* Less than 5% of renal cell carcinomas are associated with hereditary syndromes [30, 48]. In addition to those occurring in the previously described von Hippel–Lindau, hereditary papillary renal cell carcinoma and Birt–Hogg–Dubé syndromes, there are also a number of more recently recognised hereditary renal tumours.

*Succinate dehydrogenase-deficient RCC* is rare and results from inherited germline mutations in the succinate dehydrogenase (SDH) gene, most commonly SDHB but also in SDHA, SDHC and SDHD. Affected patients may also present with paragangliomas and gastrointestinal stromal tumours (GISTs). The associated RCCs may be multifocal and bilaterality occurs in around 25% of cases [49]. On microscopy, the RCCs are usually solid and are composed of cells with eosinophilic cytoplasm with distinctive cytoplasmic vacuolation and inclusions, although focal limited presence of these changes may hamper pathological recognition [50]. Intratumoral mast cells are also a common feature. Immunohistochemistry for demonstration SDHB is available, where a loss of staining is indicative of a mutation in the SDHB (most common), SDHC or SDHD genes. SDHA gene mutation can be demonstrated by additional absence of staining for SDHA. Most tumours are low grade and have a good prognosis, but those exhibiting high-grade features, sarcomatoid morphology or necrosis may show aggressive behaviour, with a high rate of metastasis up to 70% [1, 50].

Patients with RCC associated with hereditary leiomyomatosis and renal cancer syndrome have an autosomal dominant inherited germline mutation in the FH gene on chromosome 1 that encodes for fumarate hydratase [26, 30]. This syndrome is also associated with cutaneous and uterine leiomyomata, occurring at greater frequency than the associated RCCs. These tumours are high grade and often have a papillary architecture, with tumour cells having eosinophilic cytoplasm. However, the morphology may be very variable and lead to misdiagnosis, for example, as an unclassified RCC, collecting duct carcinoma or type 2 papillary RCC, particularly as they may be solitary and, therefore, not suspected to be part of a hereditary syndrome [51]. A characteristic histological feature is the presence of distinctive prominent nucleoli with perinucleolar halos, exhibiting an appearance reminiscent of cytomegalovirus inclusions [52]. On immunohistochemistry, the combination of a lack of FH expression and overexpression of *S*-(2-succino)cysteine (2SC) suggests a diagnosis of FH-deficient RCC, which can then be confirmed with molecular studies. These are highly aggressive tumours, even when of small size, and show frequent distant metastases [30, 53]. Cases of FH-deficient RCC that are not obviously associated with the hereditary syndrome have been shown to have a similar presentation and clinical course. It has, therefore, been suggested that germline testing and counselling should also be undertaken in these cases when diagnosed pathologically [54, 55].

*The tuberous sclerosis complex* is associated with mutations in the TSC1 (on chromosome 9) or TSC2 (on chromosome 16) genes encoding for hamartin or tuberin, respectively, and shows autosomal dominant inheritance with variable penetrance. A variety of associated tumour types typically involve the skin, brain, retina, heart or kidney. The most common tumours in the kidneys are multiple angiomyolipomas, but RCC also occurs rarely and at a younger age than sporadic tumours [56, 57]. The RCCs in this syndrome have been described as exhibiting three differing morphological growth patterns: the most common are RCCs with prominent smooth muscle stroma and voluminous clear cells with a tubulopapillary growth pattern, and the others resemble chromophobe-like RCCs or are similar to the sporadic eosinophilic solid and cystic RCC which is further described below [56]. The tumours show indolent behaviour. Previously undiagnosed tuberous sclerosis complex may be suspected if such tumours are seen in combination with multiple angiomyolipomas.

*Other tumour types* There are several other tumour types that have been described in recent years, but are not currently recognised as separate entities in the WHO classification. The eosinophilic solid and cystic RCC is a rare tumour predominantly occurring in adult females, with a broad age range [58, 59]. Paediatric cases have also been described [60]. It occurs sporadically and is identical to a group of tumours occurring in patients with the tuberous sclerosis complex. Morphologically it is characterised by a solid and cystic growth pattern, with constituent cells having voluminous eosinophilic cytoplasm with prominent granular stippling. The tumour is distinct in frequently showing CK20 positivity and typically a lack of expression of CA-IX and CK7. Limited molecular studies have demonstrated recurring copy number alterations and TSC1 or TSC2 mutations in the sporadic tumours [59, 61, 62]. The tumours usually show indolent behaviour, but metastatic disease has been reported [60].

A number of other tumours of which there is limited experience at present include thyroid-like follicular RCCs, RCCs with angioleiomyomatous stroma, the oncocytic RCC that occurs post-neuroblastoma treatment and RCCs exhibiting ALK gene rearrangements, monosomy 8 and TCEB1 mutations [1].

*Unclassified RCC* Approximately 5% of tumours remain difficult to categorise after thorough sampling and immunohistochemical assessment, because the tumour is purely sarcomatoid, the immunoprofile is not definitive or there are unusual or overlapping morphological features. Tumours composed of eosinophilic cells have been shown to cause particular difficulty in classification when they do not show distinctive features [63]. Such tumours are placed in the ‘unclassified’ category. This category will include both low- and high-grade tumours; therefore, pathologists are advised to describe the findings, so that it is clear in pathology reports at which end of the grading spectrum the unclassified tumour lies.

#### Challenges in tumour typing

Although most RCCs exhibit characteristic morphology that enables easy categorisation, with or without the assistance of routine immunohistochemistry, it is not uncommon for there to be difficulty in assigning a tumour type. RCCs show considerable morphological heterogeneity, with clear cells, oncocytic cells or a papillary architecture seen in a variety of renal tumours. A particularly problematic area is the separation of oncocytic tumours, where benign and malignant entities may have overlapping morphology. It is well known that distinguishing a benign renal oncocytoma from a chromophobe RCC may be difficult, particularly as fat invasion or vascular involvement, features normally associated with malignancy, does not necessarily exclude the diagnosis of an oncocytoma [64]. CK7 immunohistochemistry may help, as oncocytomas should show only focal staining whereas there is strong and diffuse staining in most chromophobe RCCs. Fluorescence in situ hybridisation (FISH) can also be utilised, as multiple chromosome abnormalities typically occur in chromophobe RCCs, but this investigation is not necessarily routinely available. Even expert renal tumour pathologists report problems in this area, with only 64% of a group of world experts willing to definitively report benign oncocytomas on biopsy samples [65].

Panels of antibodies are used in immunohistochemistry to aid classification of tumours lacking typical morphology. However, routinely available antibodies do not exclusively stain one particular tumour type, thus creating diagnostic difficulty when the immunoprofile is not conclusive. In addition, some antibody stains may be positive adjacent to areas of necrosis, such as CA-IX or CK7, thus there may be aberrant staining in partly necrotic tumours in which these antibodies are usually negative, leading to misinterpretation of results.

Furthermore, high-grade tumours, particularly in a metastatic setting, may lose characteristic morphological features and immunoprofiles, making confirmation of renal origin and tumour subtyping difficult. Pax-8 is helpful for determining renal origin, but is also positive in tumours from other sites, such as the thyroid gland and gynaecological tract tumours of Müllerian origin. Additionally, the rarity of some tumours, which may show only subtle morphological changes from the more common RCC types, makes it difficult to ensure that pathologists are able to recognise these unusual tumour types. In current practice, those tumours that do not exhibit sufficiently characteristic morphological features or immunoprofiles to allow confident categorisation into one of the known tumour types, therefore, remain ‘unclassified’.

#### Tumour grade

The Fuhrman grading system, assessing nuclear and nucleolar features, has been in international use for many years [66]. Although it has proven prognostic utility, intra- and inter-observer reproducibility has been problematic, due to difficulties in consistently applying the four grades as described morphologically [67–70]. A new WHO/ISUP grading system has been introduced following the conclusions of the 2012 ISUP Vancouver conference and is recommended for use by the WHO [1, 5]. This is also a four-grade system, with the degree of nucleolar prominence assessed to determine grades 1–3 and the presence of highly atypical ‘pleomorphic’ cells and/or sarcomatoid or rhabdoid morphology (see below) defining grade 4. The tumour grade is assigned according to the highest grade cells present, rather than the most predominant. In practice, the new WHO/ISUP grading, though similar to the Fuhrman system, is easier to apply and should be more reproducible and clinically relevant.

The WHO/ISUP grading system only applies to clear cell and papillary RCCs, as its prognostic utility has not been validated for other tumour types [67–70]. Although different grading systems have been proposed for chromophobe RCC, to date, none have been internationally accepted for use in clinical practice [71, 72]. Collecting duct carcinomas are not graded, as by definition they are aggressive high-grade tumours. Grading of RCCs should be more consistent following the introduction of the WHO/ISUP grading system. However, grading is still subject to variation depending upon the extent of sampling of tumours, which may show only focal high-grade areas, and an individual pathologist’s microscopic assessment.

It has been proposed, though not formally accepted, that tumour necrosis should be incorporated into the WHO/ISUP grading system, to further refine the prognostic significance of tumour grades, as some studies have shown that the presence or absence of necrosis influences prognosis within a specific tumour grade [73, 74]. More recently, the prognostic relevance of the different morphological patterns observed in clear cell RCC has lead to the proposal of a different grading system based on tumour architecture [75].

#### Tumour necrosis

Tumour necrosis is included in prognostic algorithms for patient management, but only histological coagulative necrosis is recognised to have prognostic significance, as grossly visible tumour necrosis is possibly due to a different mechanism (probable infarction due to the presence of tumour thrombus) [76]. Tumour necrosis has been shown to have prognostic significance for clear cell and chromophobe RCCs, independent of tumour stage and grade [5, 22, 77–79]. Papillary RCCs often contain areas of necrosis and its presence in this tumour type, therefore, lacks the same significance. However, a study of a series of type 1 papillary RCCs by Peckova et al. demonstrated a good clinical outcome for cystic and extensively necrotic tumours [80]. Necrosis has also been shown to be an adverse prognostic factor in *t*(6,11) translocation RCCs [81]. A study by Collins and Epstein of RCCs with extensive necrosis has, however, shown the situation to be more complex, with widespread cystic necrosis in high-grade tumours associated with a worse prognosis, but suggesting a good prognosis for low-grade tumours where the tumour type, grade and stage have greater prognostic significance [82]. Necrosis may also influence treatment efficacy, as, for example, the response to VEGF/tyrosine kinase inhibitor-targeted therapy has been shown to be poor in patients with metastatic disease where there was 10% or more necrosis in the primary clear cell RCC [83].

The quantity of tumour necrosis has been reported to affect the prognostic significance, not simply its presence [84–86]. However, although it has been recommended that the proportion of necrosis is recorded in histology reports, there is no international agreement as yet on how this can be reliably assessed pathologically [5].

#### Sarcomatoid and rhabdoid morphology

High-grade morphological features, such as the presence of cells with a sarcomatoid appearance, are associated with a poor outcome, with 15–22% 5-year survival reported and distant metastases commonly present at diagnosis (45–77%) [5, 87–90]. On histology, the most common pattern seen is a spindle cell sarcoma morphology; however, any pattern of sarcoma may be seen, including fibrosarcoma, chondrosarcoma and many others [91]. This appearance may be present in any of the main tumour types, occurring in approximately 5% of cases [5]. There is no minimum amount of sarcoma that needs to be seen to record this component. Tumours consisting only of sarcomatoid cells are placed in the ‘unclassified’ category in the WHO classification [1]. The extent of sarcomatoid morphology has been shown to adversely affect survival, but there is no international agreement on how this can be measured and reported reliably [92, 93].

Rhabdoid morphology refers to the presence of large atypical cells with eccentric nuclei. Its presence is also reported to be associated with a poor prognosis [94–96]. It may occur in any RCC type, but is most commonly seen in clear cell RCCs [97]. At the molecular level, an association has been shown between the rhabdoid phenotype and alterations in the switch/sucrose nonfermentable (SWI/SNF) chromatin modelling complex, similar to that noted in aggressive carcinomas from other sites exhibiting a rhabdoid or undifferentiated phenotype [98].

Sarcomatoid and rhabdoid cells may occur together and are both classified as WHO/ISUP grade 4 [1]. The presence of sarcomatoid morphology has, however, been shown to have a more significant association with death from RCC than the presence of rhabdoid morphology [99].

#### Tumour stage

Pathological tumour stage (TNM) is the most important prognostic factor for RCC and is derived from assessment of macroscopic and microscopic features [14, 100]. These include the tumour size and determination of the presence or absence of invasion into the perinephric fat, the renal sinus or Gerota’s fascia, involvement of the renal vein or inferior vena cava, direct invasion of or metastatic spread to the adrenal gland and lymph node metastases.

The 7th editions (TNM7) of the Union for International Cancer Control (UICC) and the American Joint Committee on Cancer (AJCC) TNM staging systems have been in use worldwide for over 7 years and have been validated in clinical practice [101, 102].

Recently, the 8th editions (TNM8) of the alternative UICC and AJCC staging systems were published [103, 104]. Initially, there were a number of differences between the two in a range of tumour sites, including urological tumours, but following publication of errata, the pathological TNM stage is now closely aligned for renal tumours, although significant differences still exist in the prognostic stage groupings [105, 106]. TNM8 has been recommended for use from 1st January 2018 [103, 104]. Although much of the TNM pathological staging remains unchanged in TNM8, there are a few significant changes from TNM7 which are mentioned in the relevant sections below.

*pT1 and pT2* Tumour size is part of TNM staging, with 40, 70 and 100 mm being the boundary points for increasing stage categories (pT1a, pT1b and pT2a, respectively). The maximum diameter of the tumour (excluding any intravascular extension), is assessed on macroscopic examination. For localised clear cell RCC, tumour size has been shown to correlate with clinical outcome, with increasing tumour size correlating with a worse prognosis [107]. Most tumours that exceed a maximum of 70 mm diameter show renal sinus involvement (fat or vascular invasion) and are, therefore, of higher stage [108].

*pT3* The presence of perinephric fat invasion is part of TNM staging (pT3a). The nature of the invasive edge, whether pushing (rounded) or infiltrative, has been shown to have prognostic significance, with 3-year survival rates of 75% and 27%, respectively, although specification of the growth pattern is not currently a required item for pathology reports [109].

Renal sinus invasion, defined as invasion into sinus fat/connective tissue or within vascular spaces, is the principal route of extra-renal spread of RCCs (pT3a). Through the extensive work of Bonsib, it is now recognised as a vital area for pathological assessment to ensure accurate staging [110–112]. The presence of renal sinus invasion has been shown to be associated with more aggressive tumour behaviour than perinephric fat invasion [113].

Only gross invasion of the renal vein or its ‘muscle containing segmental branches’ is included in TNM7 stage pT3a. However, for TNM8, the requirement for ‘gross’ invasion is removed, as is the need for involved branches (tributaries) of the renal vein to contain muscle. The TNM8 pT3a stage, therefore, includes the presence of any involvement of the renal vein and its branches, whether identified grossly or microscopically.

Invasion of the pelvicalyceal system has been shown in some studies, including a meta-analysis involving over 9000 patients, to be associated with poor survival and to reduce recurrence-free survival [114–117]. It is not part of TNM7 staging, but is included in TNM8 as pT3a.

pT3b is defined as involvement of the inferior vena cava (IVC) below the diaphragm and pT3c as IVC involvement above the diaphragm or if there is evidence of direct invasion of the vein wall at any level. In TNM8, the need for the IVC involvement to be visible grossly has been removed.

*pT4* The presence of direct invasion of the ipsilateral adrenal gland has a poor outcome and is TNM stage pT4 [78, 84]. It is present in 2% of cases at nephrectomy and is more often seen in large tumours, those in the upper pole or those with vascular invasion [118]. Invasion of Gerota’s fascia, taken as the surgical margin of the fat at nephrectomy, is also pT4.

*pN* Palpable lymph nodes are found in the hilar region in less than 10% of nephrectomy cases [119]. Although survival rates fall progressively as the number of lymph nodes involved by metastatic tumour increases, the TNM staging has only two categories for regional node status: pN1 for one or more positive nodes and pN0 if they are uninvolved.

*pM* pM1 is the category assigned for pathologically proven distant metastases. Adrenal metastases are stage pM1, as are nodal metastases beyond the regional lymph nodes or at other distant sites.

#### Lymphovascular invasion

Lymphovascular (microvascular) invasion (other than that within the perinephric or renal sinus fat in stage pT3a) is not part of TNM staging. However, it has been reported to correlate with survival, independent of tumour size, grade or type, and to have prognostic significance in low-stage RCC, independent of tumour grade [120–123]. Lymphatic spread to hilar lymph nodes is less often seen in clear cell RCCs than in papillary RCCs or collecting duct carcinomas.

#### Surgical margins

All surgical margins are assessed routinely in pathology, with margin involvement by tumour being generally regarded as a risk factor for local tumour recurrence. Patients with residual disease post-nephrectomy have poorer survival [124]. The presence of invasion of the renal vein wall at the surgical margin of the vein is also a risk factor for local recurrence [125]. Assessment of the parenchymal surgical margin is of importance for partial nephrectomy specimens, although one large study of over 3800 cases has shown a lack of correlation between the presence of a positive surgical margin and tumour recurrence [126].

### Potential future improvements

#### Biomarkers

There is a need to develop more reliable and readily available biomarkers for immunohistochemistry that have diagnostic, predictive and prognostic utility. Similarly, development of validated molecular tests and pathways for easy access to this advancing technology is necessary. Such tests need to be able to overcome the heterogeneity of renal tumours that affects grading and molecular characteristics in different regions of the tumour. Development of molecular tumour profiles may enable prediction of behaviour of those tumours for which the current WHO/ISUP grading system is not applicable or might obviate the need for microscopic assessment of tumour grade.

Any new tests need to be applicable to small biopsy samples. Biopsies of renal masses or metastases are becoming more frequent, being used to guide management of small renal masses and systemic treatments of metastatic disease. When an adequate sample has been achieved, renal biopsies have been shown to be highly sensitive and specific, with low complication rates [127, 128]. However, such samples, by virtue of the limited amount of tissue available and the heterogeneity of renal tumours, do pose problems with pathological diagnosis, tumour typing and assessment of grade [129, 130]. There is, therefore, a need to develop validated methods of utilising these small amounts of tumour tissue to obtain information critical for patient management.

#### Education and improved referral pathways

Many nephrectomy and biopsy specimens will be reported outside specialist centres. It is, therefore, crucial that pathologists in the wider community are able to recognise less familiar tumour types that potentially influence patient prognosis and management or have genetic implications. In the UK, for example, there is a national uropathology external quality assessment (EQA) scheme (www.histopathologyeqa.org) which has an educational role in improving standards. Pathologists are expected to participate in EQA schemes relevant to their areas of clinical practice, regularly assessing slides and receiving feedback on their performance in comparison with their peers. This type of activity and the availability of educational courses, guidelines and online resources have significantly improved knowledge of renal tumour pathology. Nonetheless, infrequent tumour types may be misdiagnosed. Referral for expert opinion is always an option, initiated by the reporting pathologist, but it is suggested that for specific cases, such as tumours occurring in younger adults, or where there is a family history or presence of multiple tumours, there should be automatic referral to a specialist centre for pathology review. This is particularly important where further FISH or molecular tests are deemed necessary.

#### Digital pathology

The advent of rapid whole-slide scanning technology, enabling the production of high-resolution and high-quality 2D and 3D digital images from conventional glass slides, has brought with it considerable opportunities for the future practice of renal tumour pathology, as well as pathology as a whole.

In clinical practice, there are the potential advantages of remote reporting and for obtaining rapid second opinions, without the need for costly and time-consuming transportation of glass slides between laboratories. Work may be distributed digitally, from centres with staff shortages, to pathologists available at geographically distant centres, thus maintaining reporting turnaround times [131]. For rarer renal tumour entities, it is possible for pathologists to share images and to obtain rapid expert opinions from around the world. Case review for multidisciplinary team meetings is facilitated by enabling images to be shared across a number of centres simultaneously. Digital macroscopic and microscopic images of specimens can also be incorporated into pathology reports and stored on hospital patient information systems.

Image analysis techniques have the potential to remove subjectivity and inter-observer variation from, for example, tumour grading, by enabling digital assessment of nucleolar size and nuclear features. The development of computer-assisted diagnostics also enables automated assessment of morphological features, either to highlight areas of interest to the pathologist, such as the highest grade areas of tumours, or to identify subtle features of prognostic significance or predictive of response to therapy [132–134].

Immunohistochemistry for biomarker evaluation in research, either on series of tumour sections or on tissue microarrays, involves assessment of the intensity and distribution of protein expression, often on a very large number of tumours. This is very laborious and time-consuming work and is subject to considerable inter- and intra-observer variation. Image analysis systems have the capability of not only speeding up analysis and handling high volumes, but may also remove the element of subjectivity inherent in manual assessment [135]. There is also potential for simultaneous assessment of multiple biomarkers on the same tissue section. Pathologist input is, however, paramount for ensuring that the correct tissue area is assessed. Such technology is also potentially advantageous in clinical practice, as the introduction of any additional biomarker assessments adds work to an already stretched specialty [136].

For clinical trials, involving large numbers of centres, there is the opportunity to centrally review digital images of tumour samples, for quality assurance, and to maintain a central image library of cases for future analysis [137].

For teaching and education, digital pathology is also highly effective. ISUP now has an image database for urological pathology, which can be accessed online (www.isup.org) and provides an invaluable resource of renal tumour images for reference and promotes standardisation of diagnosis and tumour grading [138, 139]. The capacity to annotate digital images enhances the educational value.

The wider availability of digital pathology for diagnostic use will, however, require significant investment in equipment, provision of efficient storage and retrieval facilities for the immense number of generated digital images, and for staff training. There is also a need to introduce guidelines and quality assurance checks to enable widespread use in clinical practice [140, 141]. Ultimately, it will require a culture change in the way pathology is practised in the future.

## Conclusion

In recent years, there has been considerable progress in producing guidance for pathologists in the assessment of renal tumours, thus improving consistency in diagnosis, grading and staging. The development of additional immunohistochemical and molecular tests is needed to facilitate tumour typing, because of prognostic implications, and to enable more reliable identification of poorly differentiated metastatic tumours as being of renal origin. Any new tests need to be applicable to small biopsy samples, to overcome the heterogeneity of renal tumours. There is also a need to facilitate identification of tumour types that have genetic implications. This may mean automatic referral of certain categories of patient or tumours to specialist centres or establishing national referral centres for expert review and further tests. Digital pathology has a potential role in facilitating such referral practice.

Tumour grading has established prognostic significance, but it is still based upon subjective microscopic assessment and the extent of tumour sampling. It is also not applicable to all renal tumour types. The WHO/ISUP system has advantages over the former Fuhrman grading system, but finding ways of improving assignment of grade, enhancing its relevance, or introducing an alternative means of categorising tumours is also important for future patient management.
